# Interrupting prolonged sitting reduces postprandial GIP but not GLP‐1 responses in type 2 diabetes

**DOI:** 10.1111/dom.70046

**Published:** 2025-08-22

**Authors:** Beth K. Logan, Robyn Larsen, Julian W. Sacre, Neale D. Cohen, Gavin W. Lambert, Michael J. Wheeler, Neville Owen, Bronwyn A. Kingwell, David W. Dunstan, Paddy C. Dempsey

**Affiliations:** ^1^ School of Medicine Deakin University Geelong Victoria Australia; ^2^ Baker Heart & Diabetes Institute Melbourne Victoria Australia; ^3^ Faculty of Science The University of Melbourne Melbourne Australia; ^4^ School of Health Sciences and Iverson Health Innovation Research Institute Swinburne University of Technology Hawthorn Victoria Australia; ^5^ Institute for Physical Activity and Nutrition (IPAN), School of Exercise and Nutrition Sciences, Deakin University Geelong Victoria Australia; ^6^ CSL Ltd, Bio21 Institute Melbourne Victoria Australia; ^7^ Diabetes Research Centre, Leicester General Hospital University of Leicester Leicester UK; ^8^ MRC Epidemiology Unit, Institute of Metabolic Science, University of Cambridge, Cambridge Biomedical Campus Cambridge UK

**Keywords:** GIP, GLP‐1, incretin, physical activity, sedentary behaviour, type 2 diabetes

## 1 BACKGROUND

Glucagon‐like peptide 1 (GLP‐1) and glucose‐dependent insulinotropic polypeptide (GIP) are two key intestinal‐derived incretin hormones with therapeutic potential for glycaemic and weight management.^1^ Postprandial GLP‐1 and GIP potentiate insulin secretion from pancreatic beta cells.^1^ In type 2 diabetes (T2D), although the insulinotropic effect of GLP‐1 is preserved, GIP exhibits aberrant signalling at beta cells, attenuating insulin secretion.^1^ Breaking up prolonged sitting with brief bouts of activity can improve postprandial glucose, insulin, lipids, and blood pressure responses in T2D^2^, ^3^, ^4^; however, the effect of these behaviours on incretin hormone responses in T2D remains unknown. Given the role of incretin hormones in postprandial glycaemic regulation, we examined the potential acute effects of interrupting prolonged sitting with brief bouts of light‐intensity walking (LW) and simple resistance activities (SRA) on postprandial GLP‐1 and GIP responses in middle‐aged and older females and males with T2D.

## 2 METHODS

This randomised crossover trial was conducted at the Baker Heart & Diabetes Institute (Melbourne, Australia) between October 2013 and November 2014. Full screening procedures and participant characteristics have been described previously.^2^ In brief, 24 participants (14 males, 10 females; mean ± SD age, 62 ± 6 years; body mass index, 33.0 ± 3.4 kg m^−2^; HbA1c, 7.2 ± 0.7%; estimated Glomerular Filtration Rate, 87 ± 8 mL min^−1^ 1.73 m^−2^; diabetes duration 6.8 ± 5.1 years; 23 taking metformin; 15 taking statins) completed 3 trial conditions in a random order, each separated by 6–14 days: prolonged uninterrupted sitting (SIT; control), interrupted sitting with LW and interrupted sitting with SRA. Participants were blinded to condition order until the night before the first trial condition.

### 2.1 Trial conditions

Participants arrived at the laboratory at 0715 following a 12‐hour fast. Each 8‐hour trial condition included a 1‐h steady‐state sitting period before consuming a standardised breakfast (1 h) and lunch (4.5 h). The experimental protocols commenced at the intake of breakfast as follows:

- SIT: Participants remained seated in a comfortable chair (except for lavatory breaks) and were instructed to minimise excessive movement.
- BREAKS: On separate days, participants completed two trial conditions whereby sitting was interrupted every 30 min (12 occasions, totalling 36 min) by either 3‐min bouts of LW on a treadmill (3.2 km h^−1^ with zero gradient) (LW); or by 3‐min bouts of SRA (alternating between body weight resisted half squats, calf raises, and knee raises with a gluteal contraction while following a standardised video recording) (SRA).

### 2.2 Blood sampling and laboratory analysis

Venous blood samples were collected fasted and every 30 min throughout each condition. Plasma GIP_Total_ and GLP‐1_Total_ were measured using ELISA kits (EZHGIP‐54K and EZGLP1T‐36K; Merck Millipore) in *n* = 23 participants (one female participant was excluded due to insufficient sample availability). Intra‐assay coefficients of variation were <5%.

### 2.3 Data handling and statistical analysis

Postprandial GLP‐1 and GIP responses during each trial were summarised as mean values and total and net incremental areas under the curve. Total area under the curve (tAUC) was calculated using the trapezoidal method from baseline concentration of zero. Net incremental area under the curve (iAUC) was calculated as the total incremental area below the curve, subtracting the area below each pre‐meal baseline concentration from that above. Generalised linear mixed models with random intercepts examined the differential effects of the experimental conditions on all summary outcomes using Stata 14 (Stata‐Corp LP), adjusting for potential covariates explaining residual outcome variance (age, body mass index, sex), baseline values, and period effects (treatment order). Sex‐by‐condition interactions were tested for each outcome. All models met linearity and normality of residual assumptions; statistical significance was set at *p* < 0.05.

## 3 RESULTS

**FIGURE 1:**
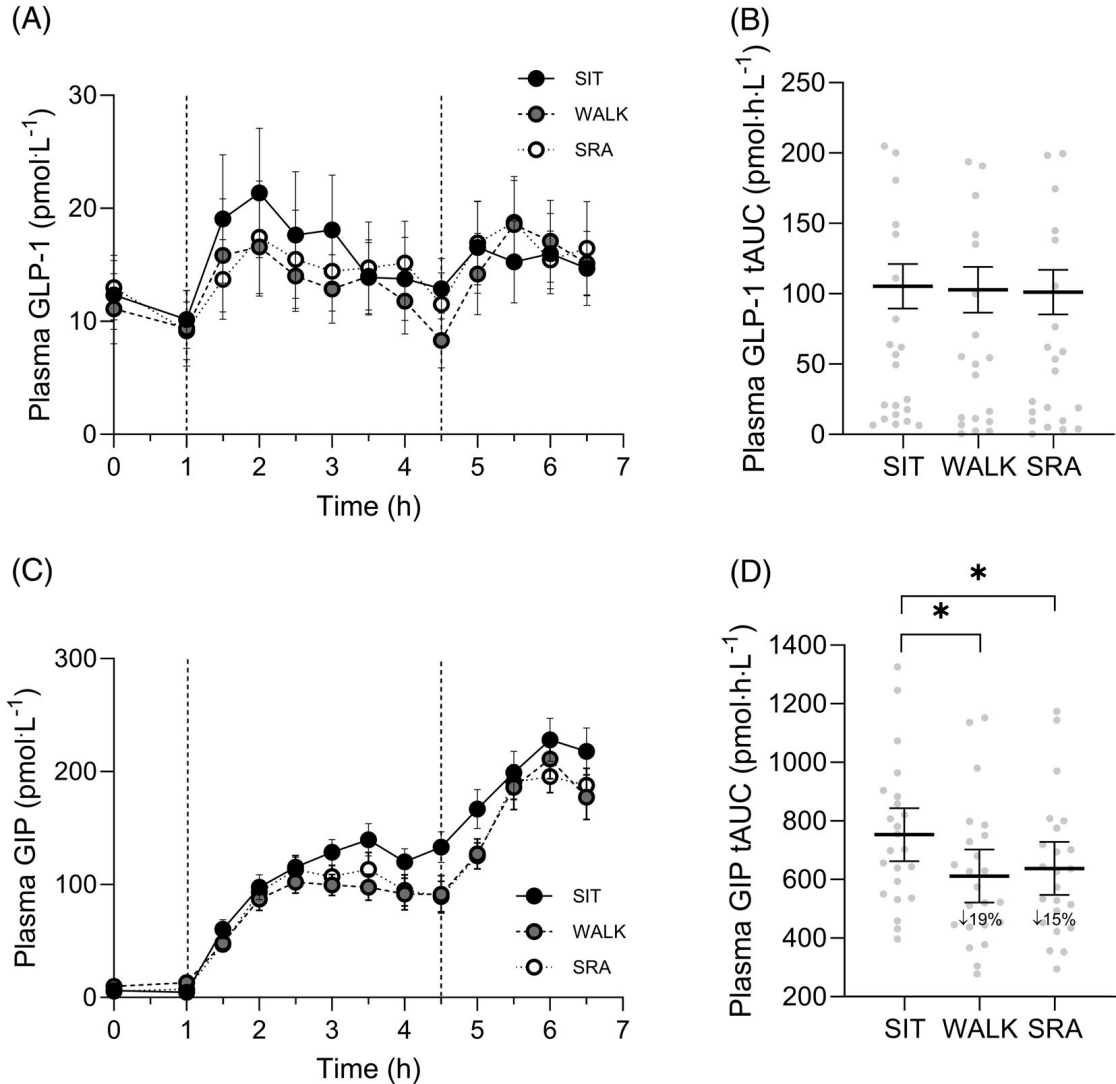
The effect of prolonged uninterrupted sitting (SIT) and sitting interrupted with 3‐min light walking (WALK) and simple resistance activity (SRA) breaks on plasma glucagon‐like peptide 1 (GLP‐1) (A) and glucose‐dependent insulinotropic polypeptide (GIP) (C) concentrations over time. Time course data are presented as marginal means ± SEM (A, C). Vertical dashed lines in (A) and (C) indicate the timing of breakfast (1 h) and lunch (4.5 h) meals. Total area under the curve (tAUC) results are presented as marginal means ± 95% CI, with underlying individual data points representing model‐adjusted (fitted) values from the mixed model (B, D). Asterisk indicates difference from SIT (*p* < 0.05).

Figure 1 shows mean plasma GLP‐1 and GIP concentrations across the three conditions (*n* = 23 participants; 14 males; 9 females). Overall (6.5 h) and meal‐specific tAUC and iAUC_net_ were significantly lower in LW and SRA versus SIT for GIP, but not for GLP‐1 (Figure 1 and Table 1). No significant differences were observed between the LW and SRA conditions.

**TABLE 1:** Overall and meal‐specific GLP‐1 and GIP concentrations between conditions.

	SIT	WALK	SRA
GLP‐1			
Overall tAUC (pmol h L^−1^)	105 [89, 121]	103 [86, 119]	101 [85, 117]
Overall iAUC_net_ (pmol h L^−1^)	37 [21, 53]	36 [19, 52]	33 [17, 49]
Mean value (pmol L^−1^)	18 [15, 21]	17 [14, 21]	17 [14, 20]
Breakfast iAUC_net_ (pmol h L^−1^)	25 [14, 36]	23 [12, 34]	20 [10, 31]
Lunch iAUC_net_ (pmol h L^−1^)	15 [8, 23]	14 [6, 22]	16 [8, 23]
GIP			
Overall tAUC (pmol h L^−1^)	753 [663, 844]	612 [521, 703]^a^	638 [548, 728]^a^
Overall iAUC_net_ (pmol h L^−1^)	707 [619, 795]	565 [476, 653]^a^	591 [503, 679]^a^
Mean value (pmol L^−1^)	147 [130, 164]	118 [101, 134]^a^	124 [107, 140]^a^
Breakfast iAUC_net_ (pmol h L^−1^)	411 [351, 471]	309 [249, 369]^a^	333 [273, 393]^a^
Lunch iAUC_net_ (pmol h L^−1^)	369 [326, 412]	304 [261, 347]^a^	308 [266, 351]^a^

*Note*: Mean values are the average values for all time points after baseline/fasting. Data are presented as marginal means ± 95% CI.

Abbreviations: CI, confidence interval; GIP, glucose‐dependent insulinotropic polypeptide; GLP‐1, glucagon‐like peptide 1; iAUC, incremental area under the curve; SIT, prolonged sitting; SRA, simple resistance activity; tAUC, total area under the curve; WALK, light walking.

^a^ Difference from SIT (*p* < 0.05).

Sex‐stratified analysis showed higher overall GLP‐1 and GIP levels in males compared to females, with a greater magnitude of GIP reduction in females in response to LW and SRA (Figure S1). However, a significant sex condition interaction (*p* < 0.05) was only observed for breakfast iAUC_net_ (LW and SRA vs. SIT; Table S1).

## 4 CONCLUSIONS

This is the first study to examine the acute effects of interrupting prolonged sitting on postprandial incretin responses in adults with T2D. We found that interrupting sitting with LW or SRA significantly reduced postprandial GIP levels compared to uninterrupted sitting (SIT). In contrast, the effects on GLP‐1 levels were more variable and not significantly different.

Previous research suggests that exercise acutely increases postprandial GLP‐1 and GIP concentrations in healthy individuals,^5^ while in T2D, a single bout of exercise did not acutely alter postprandial GIP or GLP‐1 levels.^6^ Interestingly, the use of metformin in T2D is associated with elevated total plasma GIP and GLP‐1, compared to people taking a placebo.^6^ Our findings challenge this by showing that undertaking frequent activity breaks to interrupt prolonged sitting throughout the day reduces postprandial GIP in T2D, with unclear effects on GLP‐1.

Chen et al. (2022) recently reported that in people with central adiposity (but without T2D), breaking up prolonged sitting with brief walking bouts increased postprandial GLP‐1 and had no effect on GIP.^7^ In contrast, our results in people with T2D show no change in GLP‐1 but a clear significant reduction in GIP when sitting was interrupted. This suggests a T2D‐specific dysregulation of incretin responses, in which GIP is more sensitive to activity than GLP‐1. Interestingly, lower postprandial GIP is associated with reduced cardiovascular morbidity and mortality,^8^ which may help explain some of the clinical benefits of reducing sedentary time. Consistent with previous evidence, postmenopausal females in our study had lower overall GLP‐1 and GIP concentrations than males, likely reflecting oestrogen's role in incretin secretion and glucose metabolism.^9^, ^10^

The specific mechanisms governing incretin feedback and regulation in T2D remain unclear. Because we only measured hormone concentrations, we cannot identify the precise pathways involved. Possible explanations include modulation of gastric emptying,^11^ enhanced insulin‐independent and ‐dependent skeletal muscle glucose uptake via improved insulin sensitivity,^2^, ^12^ or a negative feedback loop that suppresses GIP secretion in response to intermittent activity.

Strengths of this study include its randomised crossover design (providing control for fixed person‐specific factors and allowing a smaller sample size), a tightly controlled laboratory setting, and extended postprandial monitoring over 8 hours. Notably, this study was conducted before GLP‐1 agonists became standard adjuvant therapy in T2D, giving a clearer view of endogenous incretin responses. Key limitations include the exploratory and acute nature of the study, precluding conclusions about long‐term changes or adaptations to breaking up prolonged sitting on these secondary outcomes. Additionally, more frequent blood sampling, particularly immediately post‐meals, would have better illuminated the precise kinetics of incretin secretion, while concurrent assessment of glucagon would have also been informative.

In conclusion, our findings suggest that interrupting prolonged sitting with brief activity breaks reduces postprandial GIP, but not GLP‐1, in adults with T2D. Since breaking up prolonged sitting is known to improve postprandial glucose and insulin responses,^2^ these benefits are likely driven by enhanced insulin sensitivity and increased skeletal muscle glucose uptake, with reduced GIP as a secondary effect. However, future larger studies should explore the pathways regulating GIP in response to prolonged sitting and physical activity, and further examine the potential sex‐specific metabolic implications for T2D management.

## AUTHOR CONTRIBUTIONS

BKL analysed and interpreted the data and wrote the first draft of the manuscript; PCD conceived of and designed the research question, conducted the study, helped analyse and interpret the data and revised the manuscript for important intellectual content. NDC provided clinical support during data collection. RNL, JWS, NDC, GWL, MJW, NO, BAK and DWD assisted in the conception and design of the research question and participated in critical revision of the manuscript for intellectual content. PCD supervised the lead author. All authors approved the final version of this manuscript.

## FUNDING INFORMATION

This research was supported by the National Health and Medical Research Council (NHMRC) project grant 1081734. PCD is supported by the NHMRC Research Fellowship Scheme (#1142685).

## CONFLICT OF INTEREST STATEMENT

The authors declare no conflicts of interest.

## Supporting information


**Data S1.** Supporting Information.

## Data Availability

The data that support the findings of this study are available on request from the corresponding author. The data are not publicly available due to privacy or ethical restrictions.
