# Supplementary material for: Interrupting prolonged sitting reduces postprandial GIP but not GLP‐1 responses in type 2 diabetes
Source: Diabetes Obes Metab. 2025 Aug 22;27(11):6771–4. doi: 10.1111/dom.70046 (PMC12515788; doi:10.1111/dom.70046)
Supplement: Supplementary file 1 — Data S1. Supporting Information. [file DOM-27-6771-s001.pdf]

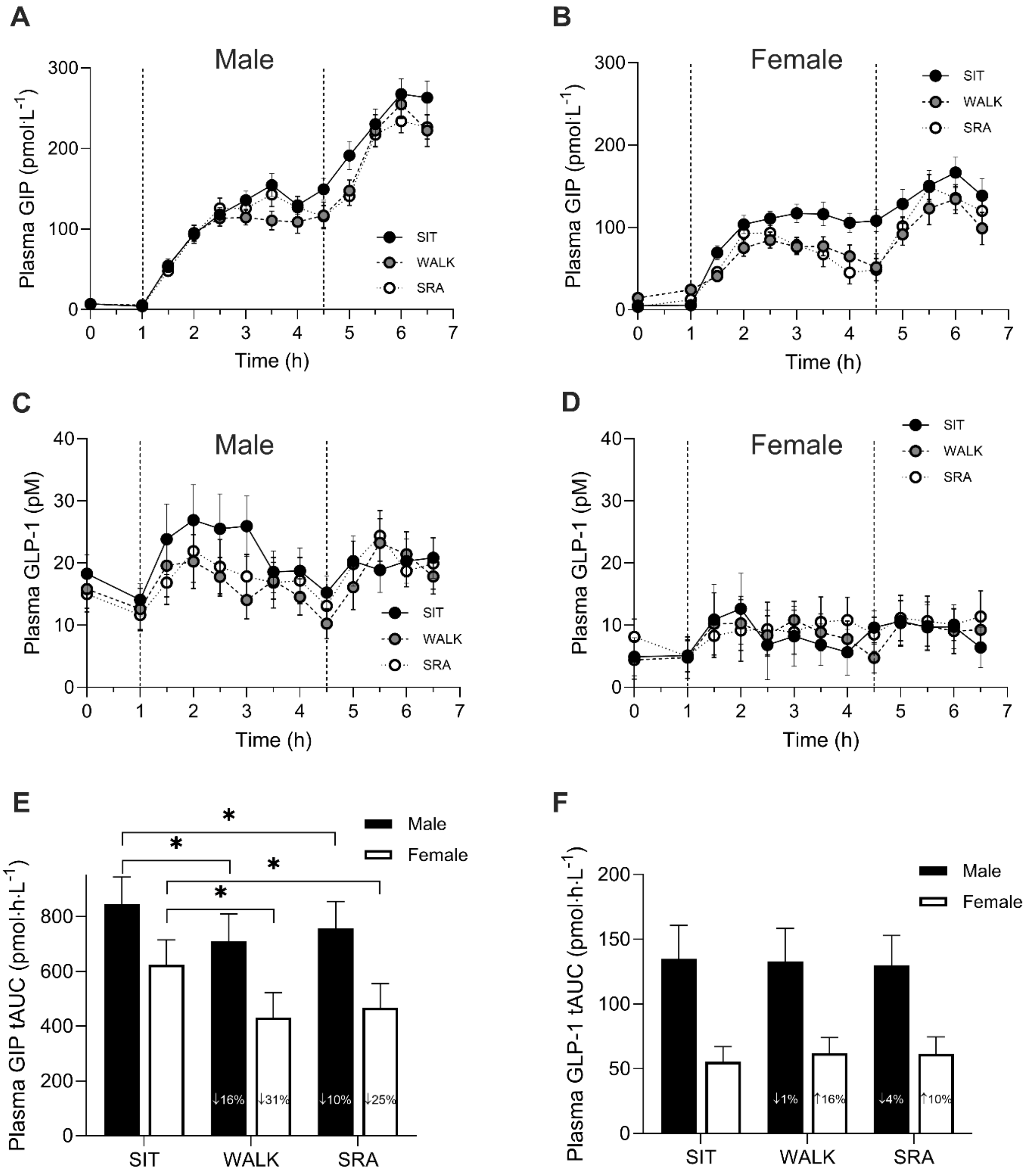

Supplementary Figure S1. Sex stratified plasma GIP (A, B) and GLP-1 concentration (C, D) between conditions. Time course data represented as marginal means ± SEM (A-D). Vertical dashed lines in time course data (panels A-D) indicate the timing of breakfast (1h) and lunch (4.5h) meals. Total area under the curve (tAUC) results are presented as marginal means ± 95% CI (E, F). *Difference from SIT (*P*<0.05).

Supplementary Table S1. Sex-stratified plasma GIP and GLP-1 concentrations between conditions and interaction effects.

	Male (n=14)			Female (n=9)			Sex interaction (male vs. female)	
	SIT	WALK	SRA	SIT	WALK	SRA	*P* (SIT vs. WALK)	*P* (SIT vs. SRA)
**GLP-1**								
Overall tAUC (pmol·h·L^{-1})	135 [109, 161]	133 [107, 158]	130 [107, 153]	55 [44, 67]	62 [50, 74]	61 [48, 75]	0.647	0.695
Overall $iAUC_{net}$ (pmol·h·L^{-1})	44 [18, 70]	42 [17, 68]	39 [16, 62]	23 [11, 34]	30 [18, 42]	28 [15, 41]	0.578	0.701
Mean value (pmol·L^{-1})	23 [18, 28]	22 [17, 27]	22 [17, 26]	10 [8, 12]	11 [9, 13]	11 [8, 13]	0.556	0.692
Breakfast iAUCnet (pmol·h·L^{-1})	28 [11, 46]	26 [9, 43]	25 [9, 40]	15 [7, 22]	22 [15, 29]	15 [8, 23]	0.441	0.837
Lunch iAUCnet (pmol·h·L^{-1})	17 [5, 30]	17 [5, 29]	17 [6, 29]	10 [4, 16]	10 [3, 16]	15 [8, 23]	0.866	0.68
**GIP**								
Overall tAUC (pmol·h·L^{-1})	845 [747, 944]	711 [612, 810]*	757 [658, 855]*	624 [534, 714]	431 [340, 522]*	466 [377, 556]*	0.169	0.213
Overall $iAUC_{net}$ (pmol·h·L^{-1})	808 [709, 906]	673 [574, 772]*	719 [621, 817]*	566 [479, 653]	369 [281, 456]*	405 [319, 491]*	0.165	0.2
Mean value (pmol·L^{-1})	165 [146, 184]	138 [119, 157]*	147 [128, 166]*	121 [105, 137]	81 [65, 98]*	90 [74, 106]*	0.149	0.223
Breakfast iAUCnet (pmol·h·L^{-1})	447 [381, 514]	362 [295, 428]*	406 [339, 472]	363 [301, 424]	212 [150, 274]*	226 [165, 287]*	0.038	0.007
Lunch iAUCnet (pmol·h·L^{-1})	444 [394, 494]	372 [323, 422]*	375 [326, 425]*	259 [215, 304]	182 [137, 227]*	212 [168, 256]	0.616	0.649

Data are marginal means ± 95% CI. SIT, prolonged sitting; WALK, light walking; SRA, simple resistance activity. *Difference from SIT (*P*<0.05).
